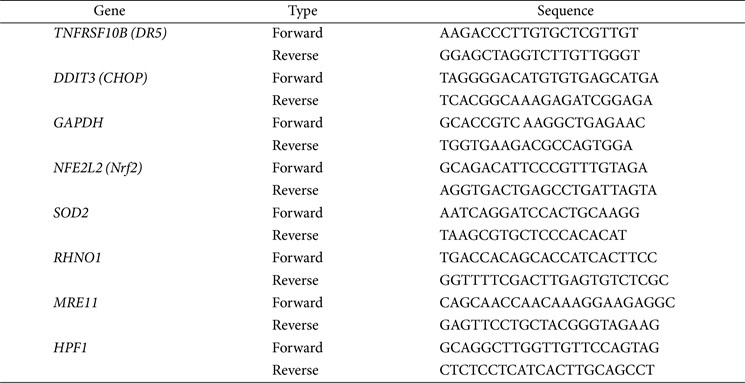# Shiga Toxin Type 2 Aggravates G1/S Phase Cell Cycle Arrest, Mediating Caspase-Independent Cell Death under Hyperosmotic Conditions in the Kidney

**DOI:** 10.4014/jmb.2505.08015

**Published:** 2025-09-05

**Authors:** So-Hyeon Park, Kyung-Soo Lee, Jun Young Park, Dae-Soo Kim, Hyun-Soo Cho, Ji Heon Noh, Chang-Ung Kim, Moo-Seung Lee

**Affiliations:** 1 Environmental Diseases Research Center, Korea Research Institute of Bioscience and Biotechnology, Daejeon 34141, Republic of Korea; 2 Department of Biochemistry, Chungnam National University, Daejeon 34134, Republic of Korea; 3 Digital Biotech Innovation Center, Korea Research Institute of Bioscience and Biotechnology, Daejeon 34141, Republic of Korea; 4 Department of Biomolecular Science, KRIBB School of Bioscience, Korea University of Science and Technology (UST), Daejeon 34113, Republic of Korea; 5 Stem Cell Convergence Research Center Korea Research Institute of Bioscience and Biotechnology, Daejeon 34141, Republic of Korea; 6 College of Pharmacy, Chosun University, Gwangju 61452, Republic of Korea

**Keywords:** Shiga toxin, hemolytic uremic syndrome, hyperosmotic condition, caspase-independent cell death, G1/S phase cell cycle arrest

## Abstract

Shiga toxin (Stx) is a virulence factor produced by *Shigella dysenteriae* serotype 1 and Stx-producing *Escherichia coli* (STEC). It causes severe renal damage, leading to hemolytic uremic syndrome (HUS). The main target organ of Stx, the kidney, plays a role in maintaining water homeostasis in the body by increasing an osmotic gradient from the cortex to the medulla. However, the activity of Stxs under kidney hyperosmotic conditions is not well understood. In this study, to investigate the Stx effects under hyperosmotic interstitial fluid, kidney epithelial cells and 3D spheroids were treated with Stx2 and NaCl. Stx2 treatment without NaCl addition increased ER stress, mitochondrial damage, reactive oxygen species, and cleaved caspase 3, 7, while co-treatment with Stx2 and NaCl showed reduced ER-mediated apoptosis. Significantly, NaCl treatment increased HSP70 expression, leading to reduced ER stress by Stx2. Furthermore, cell toxicity by Stx2 and NaCl treatment was increased by the HSP70 inhibitor. Contrariwise, DNA repair gene expression level was decreased, and G1/S phase cell cycle arrest was aggravated after being treated with Stx2 and NaCl than the Stx2 group. Importantly, lactate dehydrogenase and early/late apoptotic cell death were promoted by Stx2 under NaCl treatment. Changes in Stx2 activity at NaCl conditions were confirmed through transcriptome analysis. In conclusion, a hyperosmotic environment changes the cell death mechanism and accelerates the cytotoxicity of Stx, which suggests the importance of studying environmental factors from a pathological perspective and a therapeutic perspective at HUS.

## Introduction

Hemolytic uremic syndrome (HUS) is a life-threatening clinical condition characterized by intravascular hemolysis, thrombocytopenia, and acute renal failure [1]. HUS can be caused by genetic abnormalities, medications, and uncontrolled complement activation. However, over 90% of cases are attributed to infection with Shiga toxin-producing *Escherichia coli* (STEC) [2]. STEC-associated HUS primarily affects children under five years of age [3], with an acute mortality rate of 1–4% and long-term complications in approximately 30% of pediatric patients [4]. Importantly, antibiotic use during STEC infection is contraindicated because it induces Shiga toxin (Stx) expression, thereby increasing the risk of HUS development [5, 6]. To date, there is no specific or effective therapy for STEC-mediated HUS [7], underscoring the need for mechanistic studies that can inform future therapeutic strategies.

Stxs are classified into two major types- Stx type 1 (Stx1) and Stx type 2 (Stx2) according to amino acid similarity [8]. Both Stxs cause bloody diarrhea, HUS and central nervous system disorders [9]. However, Stx2 has been associated with more enzymatic activity and severe clinical outcomes, including weight loss, systemic toxicity, and renal injury [10-12]. After being secreted in the gut lumen during STEC infection, Stxs enter the circulation and reach target organs such as the kidney, intestine and brain [13-15]. After reaching target organs, Stx binds to its cellular receptor, globotriaosylceramide (Gb3), and undergoes retrograde transport via endocytosis to the Golgi apparatus and endoplasmic reticulum (ER) [16]. Stx is composed of one enzymatically active A subunit and five receptor-binding B subunits, forming a characteristic AB5 structure in which the B subunits are responsible for binding to the Gb3 receptor [17]. The A subunit, which possesses N-glycosidase activity, depurinates 28S rRNA and nuclear DNA, leading to translational inhibition and triggering a cascade of cellular stress responses—including ER stress, mitochondrial dysfunction, DNA damage, cell cycle arrest, and caspase-mediated apoptosis[18-24].

The kidney is a principal target organ of Stx-mediated injury and is physiologically unique in maintaining water balance via an osmotic gradient. As fluid moves from the renal cortex to the medulla, the interstitial space becomes progressively hyperosmotic due to the accumulation of sodium ions [25, 26]. This hyperosmolar environment is critical for urine concentration but also represents a stress condition for renal epithelial cells. While Stx is reported to cause ER stress and apoptosis in renal cells, it remains unclear whether its functional activities or related pathways of cell death are modified under hyperosmotic conditions. Further, while the kidney adopts protective mechanisms to prevent ER stress when it encounters changes in osmotic conditions, Stx simultaneously promotes ER stress and apoptosis, which indicates a possible mechanistic conflict under physiological conditions. Thus, in the present study, we investigated how hyperosmotic conditions influence the cytotoxic effect of Stx2 on renal epithelial cells and 3D kidney spheroids. We found that co-treatment with Stx2 and NaCl enhanced cell death predominantly through G1/S phase cell cycle arrest, but reduced ER stress and caspase-3/7 activity. Notably, NaCl treatment induced HSP70 expression, which may be a compensatory mechanism to suppress Stx2-induced ER stress. The inhibition of HSP70, nevertheless, reestablished ER stress and augmented cytotoxicity. Further, transcriptomic analysis validated that the biological activity of Stx2 was regulated by osmotic status. These results offer new perspectives into the context-dependent control of Stx2 pathogenicity and indicate that the osmotic milieu of the kidney significantly shapes the outcome of toxin-induced injury in HUS.

## Materials and Methods

### Toxins and Sodium Chloride (NaCl)

Purified Stx2 from *Escherichia coli* was purchased at List Labs (List Biological Laboratories, INC., USA). 5M Sodium chloride solution was prepared from Biosolution Co. (Biosolution, Republic of Korea) and filtered with a 0.45 micron filter. NaCl was diluted to the appropriate concentration for cell culture media.

### Cell Culture

Human proximal tubule epithelial cell line HK-2 and human renal adenocarcinoma cell line ACHN were distributed by KCLB (KCLB, Korean Cell Line Bank, Republic of Korea). HK-2 was maintained in Dulbecco’s Modified Eagle's Medium/Hams F-12 50/50 Mix (DMEM/F12; Corning, New York, USA) and ACHN was maintained in Dulbeccós Modified Eagle's Medium (DMEM; Corning, New York, USA) that containing 10% FBS and supplemented with 1% Antibiotic-Antimycotic (Thermo Fisher Scientific, USA) at 37°C with 5% CO_2_. HK-2 and ACHN cells (1.0 × 10^5^ cells/well) were seeded in 12-well plates, washed once with sterile Dulbecco’s phosphate-buffered saline (DPBS) (Sigma-Aldrich, USA), and treated with 10 ng/mL Stx2 and 25-100 mM NaCl for various time points in DMEM/F12 or DMEM containing 0.5% FBS without Antibiotic-Antimycotic (Thermo Fisher Scientific).

Frozen human mini kidney spheroids (SP-HMKS) (ScienCell Research Laboratories, USA) were thawed and resuspended in 3D-Kidney Spheroid Medium (3D-KSpM) containing 5% FBS and 3D-Kidney Spheroid Supplement (3D-KSpS). Human mini kidney spheroids were seeded into Ultra-Low Binding Culture Plates (24-well) and incubated for 24 h in a 37°C, 5% CO_2_ incubator. After 24 h, the top 70% of the cultured 3D-KSpM was replaced with fresh modified 3D-KSpM to remove residual DMSO and cultured for 3 days. After 3 days, 70% of the culture medium was replaced with PBS or NaCl-modified medium and treated with or without 30 ng/ml of Stx2. After 96 h, the phenotype of the spheroids was observed using an EVOS M5000 fluorescence microscope (ThermoFisher Scientific) and cytotoxicity was confirmed using cell culture supernatants. The 3D spheroid diameter was measured using 10 independent biological images acquired on an EVOS M5000 fluorescence microscope.

### Inhibitors

Pan-caspase inhibitor Z-VAD-FMK (Selleck Chemicals, USA) and HSP70 inhibitor VER155008 (Selleck Chemicals) in dimethyl sulfoxide (DMSO) were used 10 and 20 μM. Inhibitors were pre-added to 0.5% FBS without Antibiotic-Antimycotic (Thermo Fisher Scientific) DMEM/F12 media for 1h before being treated with or without Stx2 and NaCl.

### Cytotoxicity Assay

Cell supernatants were used to confirm cytotoxicity through lactate dehydrogenase (LDH) release. Using Pierce LDH Cytotoxicity Assay Kit, experiments were performed according to the manufacturer’s instructions. Absorbance at 490 nm and 680 nm was measured in a spectrophotometer (SpectraMAX 190 Microplate Reader; Molecular Devices, USA). The absorbance of each sample of 680 nm was subtracted from the 490 nm to exclude the background level. We performed Muse Annexin V& Dead Cell Kit (Cytek Biosciences, USA) according to the manufacturer’s protocols to evaluate apoptotic cell rates. Cells were incubated for 8 h, 24 h and 48 h and measured by Muse Cell Analyzer (Merck, Germany) after detached by Trypsin-EDTA (0.5%) (Thermo Fisher Scientific).

### Western Blot Analysis and Antibodies

All cells were harvested at each time and lysed in CETi lysis buffer (TransLab, Republic of Korea). Protein concentrations were determined by using a Pierce BCA Protein Assay Kit (Thermo Fisher Scientific). Equal amounts of (15-20 μg/lane) protein samples were divided at Bolt Bis-Tris Plus 4-12% gradient gel (Thermo Fisher Scientific) and transferred to polyvinylidene fluoride (PVDF) membrane. The membrane blocking was performed using PVDF Blocking Reagent (TOYOBO, Japan) for 1h and washed 3 times with TBST (20 mM Tris [pH 7.6], 137 mM NaCl, 0.1% Tween 20) for 5 min. After that, the PVDF membranes were incubated with proper primary antibodies at 4°C. After 24 h, PVDF membranes were washed with TBST 3 times and incubated with horseradish peroxidase (HRP)-conjugated secondary antibodies for 90 min at 21-23°C in the dark. Detection of bands was performed with Odyssey Scanner (LI-COR, USA). Primary antibodies: PERK, Phospho-PERK, IRE1α, eIF2α, Phospho-eIF2α, Bcl-2, Bax, Bak, Cytochrome C, COX IV, Cleaved Caspase-3, Cleaved Caspase-7, HSP70 (Cell Signaling Technology, USA), phospho-IRE1 (Abcam, UK). Horseradish peroxidase (HRP)-conjugated monoclonal antibody specific for human β-Actin, GAPDH. Secondary antibody: HRP-conjugated-anti-rabbit IgG (Cell Signaling Technology).

### Mitochondria Isolation

To measure the release of Cytochrome C from mitochondria to cytosol, additional mitochondria isolation was performed with Mitochondria Isolation Kit for Cultured Cells (Thermo Fisher Scientific) reference to the protocol.

### Reverse Transcription - Quantitative PCR

Total RNA was extracted by using NucleoSpin RNA Plus (Macherey-Nagel, Germany). cDNA synthesis and PCR amplification were performed with a NanoHelix RT-qPCR kit (NanoHelix, Republic of Korea). The cDNA synthesis at 50°C for 40 min and PCR Enzyme Activation at 95°C 15 min, followed by 40 cycles of denaturation at 95°C for 20 sec, annealing temperature of each primer for 30 sec, 72°C for 1 min. Fluorescence data using SYBR Green were collected at qTOWER³ (Analytik Jena, Germany). All mRNA expression data were normalized against the level of GAPDH.

### Reactive Oxygen Species Detection

Final ROS levels were observed using OxiSelect *in vitro* ROS/RNS assay kit (Cell Biolabs, USA). Cell culture supernatants were used as samples. In this assay, highly fluorescent 2’, 7’-dichlorodihydrofluorescein (DCF) is made by DCFH reacting with sample ROS/RNS. The fluorescence of DCF was measured at 480 nm excitation/ 530 nm emission by a VICTOR Nivo multimode plate reader (PerkinElmer, USA).

### Cell Cycle Analysis

For cell cycle analysis, Muse Cell Cycle Kit (Cytek Biosciences) was performed according to the manufacturer’s protocols. HK-2 and ACHN cells were incubated for 8 h, and 24 h and detached by Trypsin-EDTA (0.5%)(Thermo Fisher Scientific). Cells were washed with PBS and iced 70% ethanol was added for fixation. Fixed cells were collected by centrifugation, resuspended in cell cycle reagent and measured by Muse Cell Analyzer (Merck)[Table T1]

### High-Throughput RNA Sequencing

**RNA isolation.** Total RNA was isolated from HK-2 cells using Trizol reagent (Thermo Fisher Scientific). The quality of RNA was assessed by the Agilent 4200 TapeStation System (Agilent Technologies, USA). Qubit (Thermo Fisher Scientific) was used to perform RNA quantification.

**Library preparation and sequencing.** According to the manufacturer’s instructions, the library was constructed using QuantSeq 3' mRNA-Seq V2 Library Prep Kit FWD (Lexogen, AU). Each total RNA isolated from HK-2 cells was combined with an oligo-dT primer. The primer contained a compatible Illumina sequence at its 5’ end. After that, reverse transcription was performed. RNA templates were removed and synthesis of the second strand was started by a primer that possesses an Illumina -compatible linker sequence at the 5’ end. Using magnetic beads to eliminate reaction components, a double-stranded library was purified. To add the complete adapter sequences, amplification of the library was conducted which was required for cluster generation. High-throughput sequencing was performed as single-end 75 bp sequencing by NextSeq 500 / 550 (Illumina, USA.) using a final library that was isolated from PCR components.

**Data analysis.** Sequencing quality control of raw data was performed using FastQC [27]. Reads were sequenced and then reduced for adapter sequences and lower quality. Using STAR, the clean reads were mapped to the genome [28]. HTSeq-count was used for the quantification of reads [29]. Based on the TMM+CPM normalization method using the Python “conorm” package, the Read Counts were processed [30]. Data mining was performed using ExDEGA (Ebiogen Inc., Republic of Korea) and graphic visualization was performed using ExDEGA and Metascape [31]. Metascape uploaded data were visualized to Gene Ontology (GO). According to GO, Kyoto Encyclopedia of Genes and Genomes (KEGG) pathway analyses were performed.

### Statistical Analysis

Data were expressed as means ± SEM and analyzed using GraphPad Prism version 5.00. (GraphPad Software, USA). Values of *p < 0.05 were considered statistically significant assessed by Student's *t*-test.

## Result

### Stx2 Increases Cell Death under Hyperosmotic Conditions

**Fig. 1:**
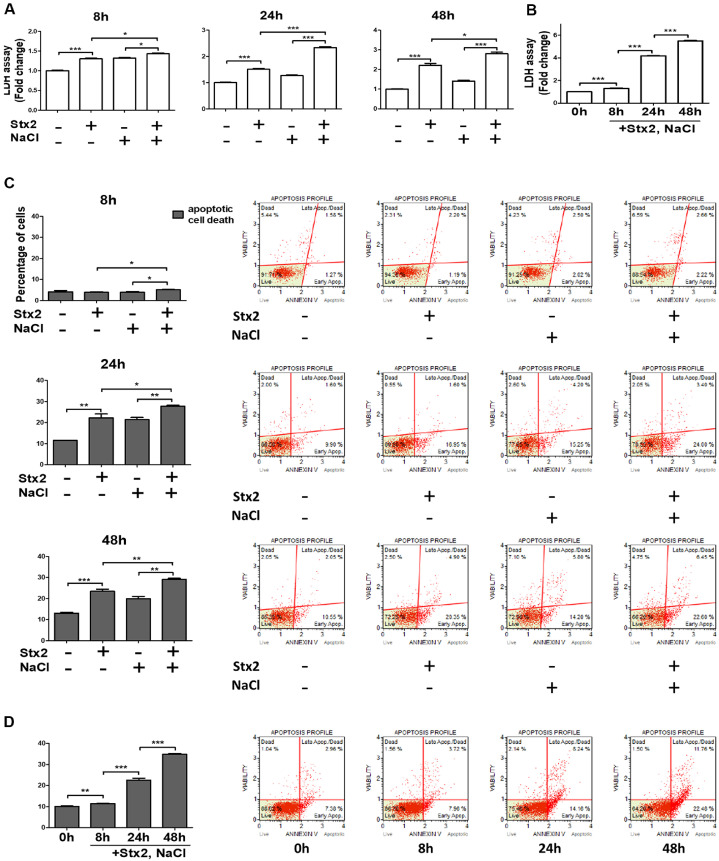
Stx2-induced cytotoxicity is enhanced under hyperosmotic conditions as assessed by LDH release and Annexin V staining. HK-2 cells were treated with Shiga toxin 2 (Stx2; 10 ng/ml) under either isotonic (unmodified) or hyperosmotic media conditions, the latter achieved by supplementing culture medium with NaCl (100 mM) to mimic the osmolarity (~500 mOsm/l) of the renal juxtamedullary interstitium. (**A**) LDH release was quantified at 8, 24, and 48 h post-treatment. Stx2 induced cytotoxicity under both conditions, with significantly elevated LDH levels observed under hyperosmotic conditions. (**B**) Time-dependent increase in LDH release was further enhanced by cotreatment with Stx2 and NaCl. (**C**) Early and late apoptotic cell death was assessed using an Annexin V/dead cell assay. NaCl alone and in combination with Stx2 increased late apoptotic cell populations. (**D**) Total apoptotic cell death (early + late) was significantly elevated at 8, 24, and 48 h following (Stx2+NaCl) treatment compared to Stx2 alone. All data represent the mean ± SEM from three independent experiments. Statistical comparisons were performed using two-tailed Student’s *t*-tests. *p < 0.05; **p < 0.01; ***p < 0.001.

It has been well documented that Stx2, as a cytotoxin, induces cell death [11]. To examine whether hyperosmotic stress potentiates Stx2-induced cytotoxicity, we treated human kidney epithelial HK-2 cells with NaCl to mimic a hyperosmotic environment. In isotonic conditions, Stx2 alone increased lactate dehydrogenase (LDH) release, indicating membrane damage. Notably, co-treatment with Stx2 and NaCl (hereafter referred to as [Stx2+NaCl]) significantly elevated LDH release at all measured time points compared to Stx2 alone (Fig. 1A). Moreover, LDH levels increased progressively over time in the [Stx2+NaCl] group, indicating a time-dependent cytotoxic effect (Fig. 1B). Flow cytometry analysis further revealed that Stx2 alone induced both early and late apoptotic cell death at 8, 24, and 48 h (Fig. 1C). Importantly, [Stx2+NaCl] co-treatment significantly augmented apoptotic cell death in a time-dependent manner (Fig. 1D). Collectively, these results demonstrate that hyperosmotic conditions exacerbate Stx2-induced cytotoxicity, suggesting that the kidney’s hyperosmotic microenvironment may augment Stx2 pathogenicity.

### Stx2-Mediated ER Stress Is Attenuated under Hyperosmotic Conditions

**Fig. 2:**
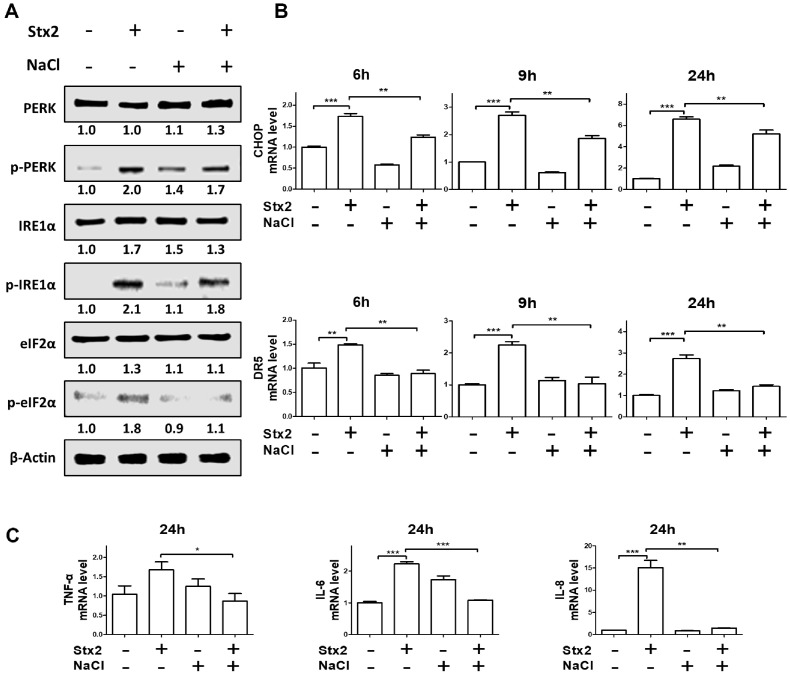
ER Stress Responses under Stx2 and NaCl Co-Treatment Assessed by Western Blot and RT-qPCR. HK-2 cells were treated with Stx2 (10 ng/ml) in the presence or absence of NaCl (100 mM) to simulate hyperosmotic conditions. (**A**) Western blot analysis revealed that Stx2 induced phosphorylation of PERK, IRE1α, and eIF2α at 24 h under isotonic conditions, while this induction was attenuated under hyperosmotic (NaCl-supplemented) conditions. (**B**) mRNA levels of ER stressrelated genes CHOP and DR5 were measured by RT-qPCR at 6, 9, and 24 h. Co-treatment with NaCl and Stx2 resulted in significantly lower CHOP and DR5 expression compared to Stx2 alone. GAPDH was used as the internal control (**C**) Expression levels of pro-inflammatory cytokines TNF-α, IL-6, and IL-8 were also assessed by RT-qPCR. NaCl co-treatment suppressed the upregulation of these cytokines induced by Stx2. All data represent three independent experiments and are presented as mean ± SEM. Statistical analysis was performed using a two-tailed Student’s *t*-test. *p < 0.05; **p < 0.01; ***p < 0.001.

As shown in Fig. 1, [Stx2+NaCl] resulted in greater cell death compared to Stx2 treatment alone. While Stx2-induced apoptotic cell death is commonly mediated by endoplasmic reticulum (ER) stress [19], we investigated whether this pathway contributes to cell death under hyperosmotic conditions. In HK-2 cells, Stx2 treatment significantly increased the phosphorylation of key ER stress sensors PERK, IRE1α, and eIF2α after 24 h (Fig. 2A). Interestingly, [Stx2+NaCl] treatment markedly reduced the phosphorylation of these ER stress markers compared to Stx2 alone. Consistent with this, mRNA levels of CHOP and DR5, which are canonical downstream effectors of ER stress, were significantly lower in the [Stx2+NaCl] group than in the Stx2-only group between 6 and 24 h (Fig. 2B). In particular, DR5 expression in [Stx2+NaCl]-treated cells was reduced to levels comparable to the untreated control group. Furthermore, pro-inflammatory cytokines including tumor necrosis factor-alpha (TNF-α), interleukin-6 (IL-6), and IL-8, which are typically upregulated in response to ER stress were also diminished in the [Stx2+NaCl] group relative to Stx2 alone (Fig. 2C). Together, these observations indicate that hyperosmotic conditions mitigate Stx2-induced ER stress and associated inflammatory responses.

### Hyperosmotic Conditions Reduce Mitochondrial Damage and Caspase-Dependent Cell Death Induced by Stx2

**Fig. 3:**
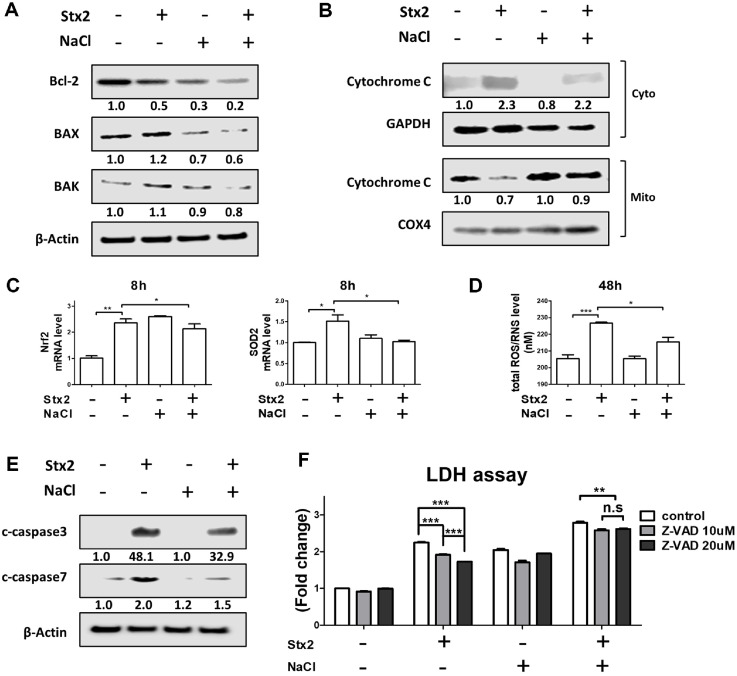
Stx2 induces mitochondrial damage and apoptosis in HK-2 cells under hyperosmotic conditions. HK-2 cells were treated with Stx2 (10 ng/ml) and NaCl (100 mM) to simulate renal hyperosmolarity. (**A**) Protein levels of antiapoptotic (Bcl-2) and pro-apoptotic (Bax and Bak) markers were assessed by western blot after 24 h. (**B**) Western blot analysis of cytochrome c in cytosolic and mitochondrial fractions was performed following organelle isolation. GAPDH and COX4 were used as cytosolic and mitochondrial loading controls, respectively. (**C**) RT-qPCR was used to assess the expression of oxidative stress-associated genes Nrf2 and SOD2 at 8 h. GAPDH served as the internal control. (**D**) Total ROS/RNS levels in the culture supernatant were measured at 48 h. (**E**) Cleavage of caspase-3 and -7 was evaluated by western blot, normalized to β-actin. (**F**) HK-2 cells were pretreated with the pan-caspase inhibitor Z-VAD (20 μM) for 1 h prior to [Stx2+NaCl] treatment. Cell death was quantified by LDH release. All data are presented as mean ± SEM from three independent experiments. Statistical significance was assessed using two-tailed Student’s *t*-tests. *p < 0.05; **p < 0.01; ***p < 0.001.

Stx2 is known to induce ER stress, which can lead to mitochondrial damage, activation of caspases, and ultimately apoptosis [19, 22]. Despite the enhanced overall cytotoxicity observed with [Stx2+NaCl] treatment (Fig. 1), we explored whether mitochondrial damage and caspase activation were still central to cell death under hyperosmotic conditions. Stx2 treatment increased the expression of pro-apoptotic proteins BAX and BAK level while reducing the anti-apoptotic protein Bcl-2 (Fig. 3A). However, [Stx2+NaCl] treatment resulted in a lower expression of BAX and BAK. Additionally, [Stx2+NaCl] reduced the cytosolic release of cytochrome c from mitochondria, suggesting attenuation of mitochondrial membrane permeabilization (Fig. 3B). Given the link between mitochondrial damage and oxidative stress [19, 22], we next investigated reactive oxygen species (ROS) production and caspase activation. Stx2 treatment upregulated mRNA levels of oxidative stress markers including Nrf2 and SOD2 (Fig. 3C), and increased total ROS/RNS in the culture supernatant, as well as caspase-3 and -7 cleavage (Fig. 3D and 3E). Notably, [Stx2+NaCl] treatment reduced ROS/RNS levels and caspase-3/7 activation compared to Stx2 alone. To further examine the involvement of caspases, we used the pan-caspase inhibitor Z-VAD-FMK. While Z-VAD-FMK significantly reduced Stx2-induced cell death, it had a diminished effect in the [Stx2+NaCl] group (Fig. 3F), indicating that during hyperosmotic stress, cell death occurs via caspase-independent mechanisms. These results show that hyperosmotic stress modifies the pathway of Stx2-induced cell death by minimizing mitochondrial impairment and caspase activation, thereby indicating a diversion toward other cell death mechanisms that are functional under physiological conditions in the kidney.

### HSP70 Induction under Hyperosmotic Conditions Attenuates Stx2-Mediated ER Stress and Cytotoxicity

**Fig. 4:**
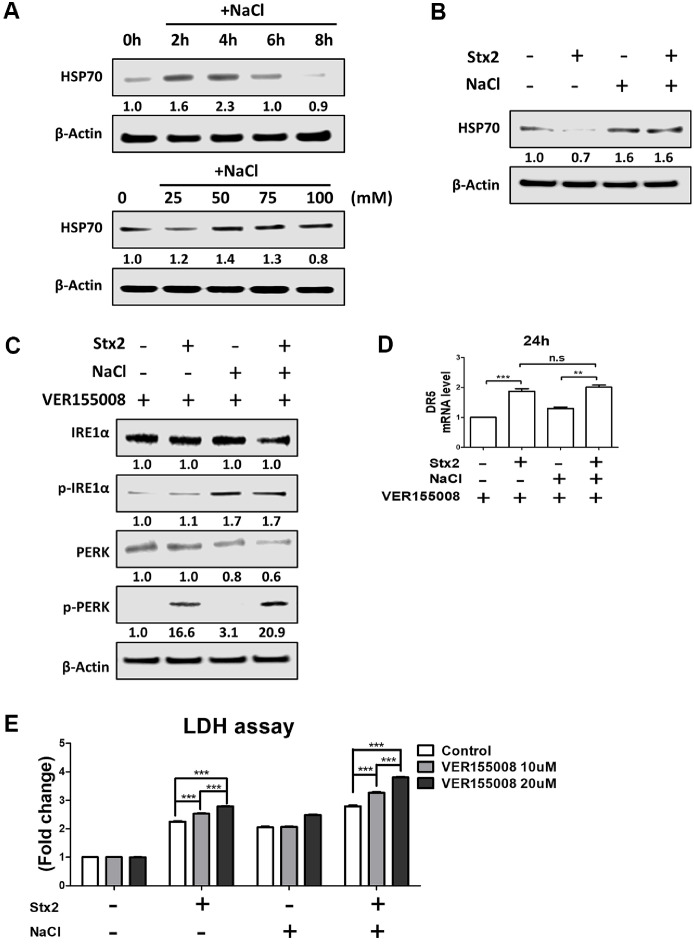
NaCl-induced HSP70 protects against Stx2-mediated cytotoxicity in HK-2 cells. (**A**) HSP70 expression was assessed by western blot at 4 hours following 100 mM NaCl treatment and after 6 h of dose-dependent NaCl stimulation (25, 50, 75, 100 mM). (**B**) HK-2 cells were treated with or without Stx2 (10 ng/ml) and NaCl (100 mM) for 6 h. HSP70 protein expression was analyzed via western blot. (**C**) Cells were pretreated with the HSP70 inhibitor VER155008 (20 μM) for 1 h, then treated with Stx2 and NaCl. Phosphorylated IRE1α (p-IRE1α) and Phosphorylated PERK (p-PERK) expression were evaluated by Western blot at 24 h (**D**) DR5 mRNA expression was measured by RT-qPCR at 24 h. (**E**) LDH release was quantified after Stx2 or [Stx2+NaCl] treatment in the presence or absence of 1-h VER155008 pretreatment. All results represent the mean ± SEM from three independent experiments. Statistical comparisons were performed using two-tailed Student’s *t*-tests. ***p < 0.001.

It is well established that heat shock proteins (HSPs) function as molecular chaperones that protect cells from various forms of stress, including hyperosmolarity, thermal shifts, hypoxia, and mechanical pressure [32, 33]. Among them, HSP70 is the most abundantly expressed isoform in the kidney and is known to play a cytoprotective role in multiple renal injury models [34, 35]. Given that ER stress is a key mechanism of Stx2-induced cytotoxicity, and that hyperosmotic conditions may engage compensatory stress responses, we investigated whether NaCl-induced HSP70 expression modulates Stx2 toxicity. NaCl treatment potently induced HSP70 expression in HK-2 cells in a dose-dependent manner, with peak expression observed at 4 h (Fig. 4A). Notably, Stx2 treatment under isotonic conditions reduced HSP70 levels, whereas [Stx2+NaCl] co-treatment preserved HSP70 expression (Fig. 4B). To determine whether HSP70 plays a functional role in modulating ER stress, we co-treated cells with the HSP70 inhibitor VER155008. Inhibition of HSP70 under [Stx2+NaCl] conditions resulted in increased levels of phosphorylated IRE1α, PERK and DR5 at 24 h, which are established markers of ER stress (Fig. 4C and 4D). In addition, inhibition of HSP70 greatly increased Stx2- and [Stx2+NaCl]-mediated cell death (Fig. 4E). Together, these results suggest that hyperosmotic stress induces HSP70 as an adaptive response, which in turn suppresses ER stress signaling and mitigates Stx2-induced cytotoxicity. Thus, HSP70 may serve as a key modulator of toxin sensitivity under physiological osmotic gradients in the kidney.

### Stx2 Aggravates G1/S-Phase Cell Cycle Arrest via Impaired DNA Repair under Hyperosmotic Conditions

**Fig. 5:**
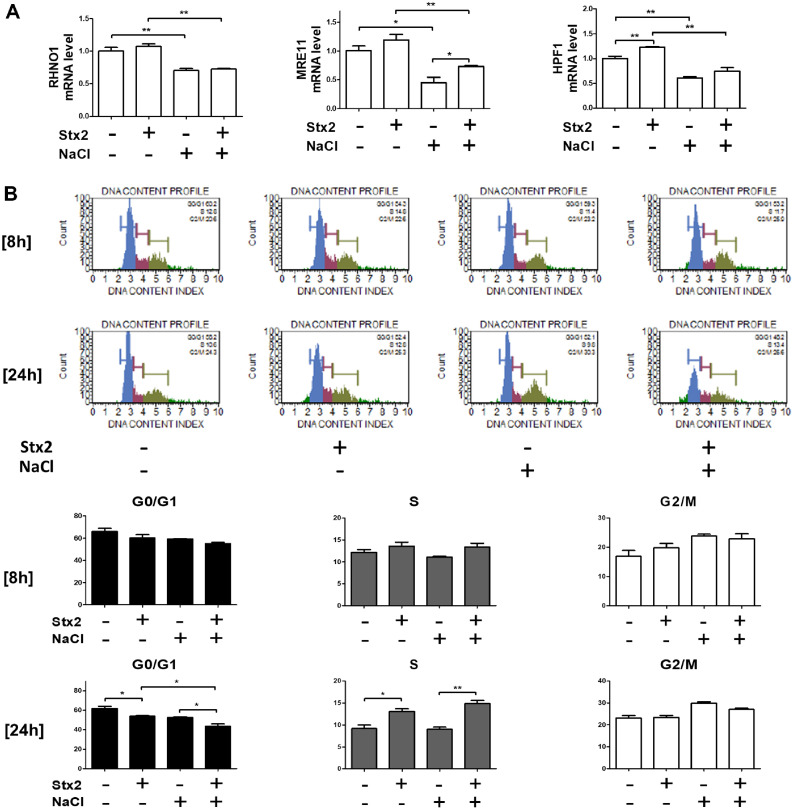
Stx2 suppresses DNA repair and exacerbates G1/S-phase cell cycle arrest under hyperosmotic conditions. HK-2 cells were cultured under isotonic (unchanged) or hyperosmotic (100 mM NaCl) conditions in the presence or absence of Stx2; 10 ng/ml for 8 or 24 h. (**A**) RT-qPCR analysis for DNA repair genes like RHNO1, HPF1, and MRE11 revealed decreased mRNA expression following co-treatment with Stx2 and NaCl at 8 h. (**B**) Cell cycle distribution was analyzed by flow cytometry as the percentage of cells in each phase. Co-treatment with Stx2 and NaCl caused a greater reduction in the G0/G1 phase than Stx2 alone, indicating increased G1/S-phase arrest. Values are presented as mean ± SEM of three independent experiments. Statistical significance was assessed by two-tailed Student's *t*-tests. *p < 0.05; ***p < 0.001.

Stx2 is known to disrupt cell cycle progression by inhibiting protein synthesis and DNA damage, leading to G1/S-phase arrest [24]. In parallel, hyperosmotic conditions have also been reported to cause DNA damage and fail to repair DNA damage [36]. Therefore, to investigate whether the enhanced cytotoxicity observed in the [Stx2+NaCl] group was associated with disrupted DNA repair and altered cell cycle progression, we examined the expression of key DNA repair genes and performed cell cycle analysis. Expression of critical DNA repair regulators, including RAD9-HUS1-RAD1 Interacting Nuclear Orphan 1 (RHNO1), Meiotic Recombination 11 (MRE11), and Histone PARylation Factor 1 (HPF1), was markedly suppressed under NaCl treatment and [Stx2+NaCl] group (Fig. 5A). Although no significant changes in cell cycle distribution were observed at 8 h, both Stx2 and [Stx2+NaCl] treatment led to a notable shift from G0/G1 to S phase at 24 h (Fig. 5B). Importantly, the [Stx2+NaCl] group exhibited a further reduction in G0/G1 phase cells compared to the Stx2-only group, suggesting exacerbation of G1/S-phase transition. These results indicate that Stx2 promotes G1/S-phase cell cycle arrest under hyperosmotic conditions by impairing the expression of DNA repair machinery, thereby contributing to enhanced cytotoxicity.

### Stx2-Induced Cytotoxicity under Hyperosmotic Conditions Is Exacerbated in Kidney Epithelial Cells and Three-Dimensional Human Mini-Kidney Spheroids

**Fig. 6:**
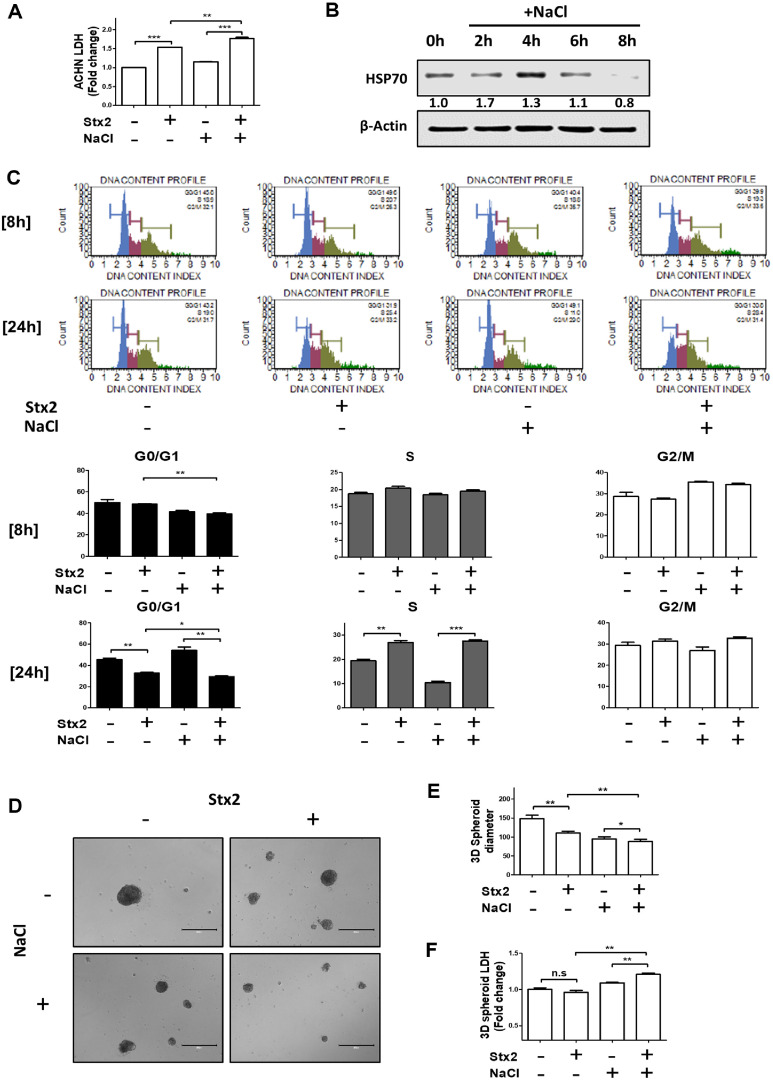
Stx2 induces kidney cell injury under hyperosmotic conditions in ACHN cells and 3D human mini kidney spheroids. (**A**) ACHN cells were incubated with unmodified and modified media (100 mM NaCl) with or without Stx2 (10 ng/ml) and enhanced LDH level was measured at 48 h. (**B**) HSP70 expression level was elevated until 4 h at ACHN cells under 100 mM NaCl (**C**) ACHN cells were collected and cell cycle phase was indicated by ratio at 8 h and 24 h. Data was collected with three independent experiments. (**D**) Representative images of three-dimensional human mini kidney spheroids showing morphological changes after Stx2 (30 ng/ml) treatment under NaCl (100 mM) unmodified and modified media for 96 h. (**E**) Quantification of spheroids diameters after Stx2 (30 ng/mL) treatment under NaCl (100 mM) unmodified and modified media for 96 h was performed with 10 independent spheroids (Biological replicates). (**F**) Elevated LDH induced by Stx2 (30 ng/ml) under unmodified and modified media (100 mM NaCl) was examined at three-dimensional human mini kidney spheroids. All data were expressed as means ± SEM. Statistical analysis was performed using two-tailed Student’s *t*-test *p < 0.05; **p < 0.01; ***p < 0.001.

The kidney comprises multiple cell types that are highly susceptible to Stxs [37]. Among them, the ACHN renal epithelial cell line has been widely used as a model in Stx-related research [38]. Additionally, three-dimensional (3D) human mini-kidney spheroids have been shown to better recapitulate *in vivo* renal physiology and pathogenesis than conventional two-dimensional (2D) cultures [39]. To evaluate whether Stx2-induced toxicity is similarly enhanced under hyperosmotic conditions in other renal models, we assessed cytotoxic effects in both ACHN cells and 3D mini-kidney spheroids, which have been validated as relevant human analogs [39, 40]. In ACHN cells, Stx2 treatment under isotonic conditions increased LDH release, which was further augmented by co-treatment with NaCl ([Stx2+NaCl]) (Fig. 6A). NaCl alone also induced HSP70 expression in a time-dependent manner, with peak levels observed at 4 h under 100 mM NaCl (Fig. 6B). Cell cycle analysis revealed that [Stx2+NaCl] co-treatment significantly reduced the G0/G1 phase population at both 8 and 24 h compared to Stx2 alone, while promoting G1/S transition at 24 h (Fig. 6C). Microscopic evaluation of 3D kidney spheroids demonstrated severe structural disruption in the [Stx2+NaCl] group, including collapse of the spheroid architecture (Fig. 6D). Consistently, spheroid diameter was significantly reduced in the co-treatment group compared to either Stx2 or NaCl alone (Fig. 6E). Moreover, while Stx2-induced LDH release was minimal under isotonic conditions, it was markedly elevated under hyperosmotic stress, indicating enhanced cytotoxicity (Fig. 6F). Thus, these findings suggest that Stx2-mediated kidney injury is exacerbated by hyperosmotic conditions, reinforcing the importance of considering renal micro environmental factors in the pathogenesis of HUS.

### Transcriptomic Analysis Reveals Distinct Stx2-Mediated Renal Cellular Damage under Hyperosmotic Conditions

**Fig. 7:**
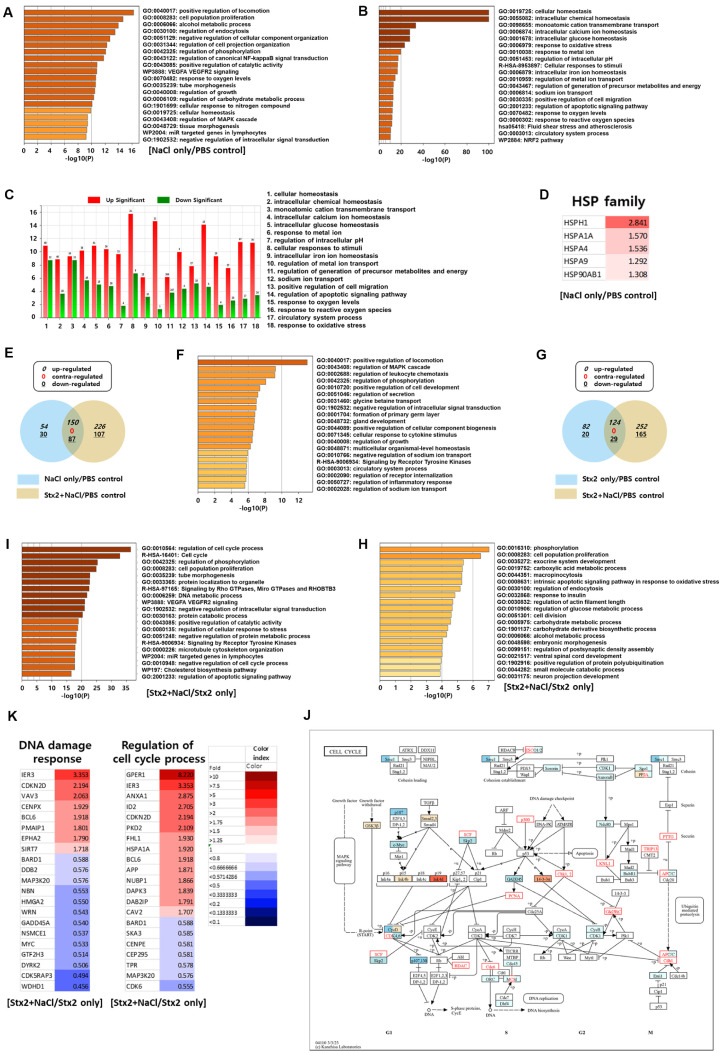
Transcriptomic profiling reveals distinct cellular responses to Stx2 and NaCl co-treatment. Differentially expressed genes (DEG) analysis was performed from two separate experiments (*n* = 2). Group 1, Group 2, Group 3, and Group 4 represent (PBS treatment group), (Stx2 treated group), (NaCl treatment group), and (Stx2 and NaCl treatment groups), respectively. (**A**) Group 3 was filtered based on fold change 1.5, normalized data (log2) 4.00 and *p*-value 0.050 and conducting enrichment analysis. (**B**) GO of [cellular homeostasis] category genes were filtered based on fold change 1.2, normalized data (log2) 4.00 and *p*-value 0.050 and conducting enrichment analysis. (**C**) Genes were filtered based on fold change 1.2, normalized data (log2) 4.00 and *p*-value 0.050 and Filter Gene Category Chart was shown. (**D**) HSP gene families were filtered based on normalized data (log2) 4.00 and *p*-value 0.050 and represented by heatmap. (**E**) Venn diagram of overlapping DEGs comparing Group 3 and 4. Genes were filtered based on fold change 2, normalized data (log2) 4.00 and *p*-value 0.050. (**F**) Conducting enrichment analysis with up-regulated genes at overlapping part of the Venn diagram Fig. 7E. (**G**) Venn diagram of overlapping DEGs comparing Group 2 and 4. Genes were filtered based on fold change 2, normalized data (log2) 4.00 and *p*-value 0.050. (**H**) Conducting enrichment analysis with down-regulated genes at Group 4 non-overlapping part (yellow) of the Venn diagram Fig. 7G. (**I**) Fold change of Group 4 based on Group 2 conducting enrichment analysis. Genes were filtered based on fold change 1.2, normalized data (log2) 4.00 and *p*-value 0.050. (**J**) Fold change of Group 4 based on Group 2 conducting Kyoto Encyclopedia of Genes and Genomes (KEGG) analysis about cell cycle pathway. Genes were filtered based on fold change 1.2, normalized data (log2) 4.00 and *p*-value 0.050. (**K**) Genes were filtered based on fold change 1.7, normalized data (log2) 4.00 and p-value 0.050. GO of [DNA damage response] and [Regulation of cell cycle process] were represented by heat map.

In an attempt to clarify the molecular mechanisms underlying Stx2-induced renal injury under hyperosmotic conditions, we carried out high-throughput RNA sequencing followed by differential gene expression (DEG) analysis in HK-2 cells. Using the Metascape pathway enrichment tool, we observed that the single treatment of NaCl (Group 3) appreciably changed cellular homeostatic genes (Fig. 7A). The genes related to homeostasis were subsequently categorized by Gene Ontology (GO) terms and revealed a general upregulation of the regulative genes of importance in cell stress adaptation, as depicted in ExDEGA (Fig. 7B and 7C). Heat shock proteins were significantly upregulated among these genes, as indicated by the heatmap (Fig. 7D). To compare Stx2 and NaCl combined transcriptional change specifically, a Venn diagram generated by ExDEGA revealed overlap between Group 3 (NaCl-treated) and Group 4 (Stx2+NaCl) (Fig. 7E). [Multicellular organismal-level homeostasis] category genes were common in both upregulated groups (Fig. 7F). Conversely, comparing [Stx2+NaCl] group (Group 4) with Stx2-only group (Group 2) revealed the evident downregulation of genes involved in the [intrinsic apoptotic signaling pathway in response to oxidative stress], indicating the inhibition of caspase-mediated cellular apoptosis in the condition of hyperosmotic stress (Fig. 7G and 7H). Further DEG fold-change value comparison (Group 4 vs. Group 2) with Metascape revealed the significant enrichment of [cell cycle process] and [cell cycle] GO terms (Fig. 7I). Pathway mapping by KEGG demonstrated that most of the altered genes were involved in the G1/S transition phase (Fig. 7J). Furthermore, the combined treatment of NaCl and Stx2 also affected the transcription of major pathways involved in [DNA damage response] and [regulation of cell cycle process], thereby further confirming the impact of hyperosmotic stress on cell cycle regulation (Fig. 7K). Therefore, these transcriptomic results indicate that a hyperosmotic environment reshapes cellular stress responses by inducing homeostasis-related genes, inhibiting apoptosis caused by oxidative stress, and enhancing G1/S-phase cell cycle arrest. This alteration in cell death programming may be responsible for the elevated cytotoxicity of Stx2 during kidney-relevant osmotic conditions.

## Discussion

HUS due to STEC infection is primarily the result of the cytotoxic effect of Stxs, particularly Stx2 [2, 41]. Thus, Stx-mediated HUS development and cellular toxicity are being investigated [9]. While the molecular mechanisms of Stx-induced cell death have been clearly explained, *in vitro* models to date cannot truly replicate the kidney's physiological microenvironment, particularly its hyperosmolarity [19-22]. In the present work, we filled this gap by adding NaCl to reproduce the hyperosmotic interstitial environment of the renal medulla according to the Perez-Pinera, Pablo *et al*. research [42, 43]. Our data indicate that hyperosmotic stress significantly affects the cellular response to Stx2, both cytotoxicity and cell death mechanisms. Under hyperosmotic conditions, we found marked augmentation of HSP70 expression, a cytoprotective chaperone expressed at high levels in kidney epithelial cells [34, 35]. HSP70 induction suppressed ER stress and downstream caspase-mediated apoptotic signaling under conditions typically induced by Stx2 (Figs. 2, 4, 6B). However, co-treatment of Stx2 and NaCl increased G1/S phase cell cycle arrest and overall cytotoxicity (Figs. 1, 5, 6C, 6F), even despite reduced ER stress. These findings suggest an alteration in the Stx2-induced mode of cell death under hyperosmotic conditions, revealing the complex dynamics of environmental stressors and cellular mechanisms of death.

HSP70 plays a central role in the regulation of ER homeostasis and apoptosis in renal pathophysiology [44-46]. Although ER stress-induced apoptosis is an established pathway of Stx-induced cytotoxicity [11, 19], the role of HSP70 in the regulation of this pathway under hyperosmotic conditions was not clear. The present study demonstrates that HSP70 expression induced by hyperosmoticity attenuates Stx2-induced ER stress and mitochondrial damage, resulting in decreased activation of caspase-3 and -7 (Fig. 3). Furthermore, pharmacological inhibition of HSP70 reversed these protective effects, defining its regulatory role under high-salt conditions (Fig. 4E). These findings underscore a previously understated mechanism by which the renal microenvironment controls toxin sensitivity. Notably, the observed cell mortality increase under [Stx2+NaCl] conditions was despite inhibitions of caspase activation, representing a switch to caspase-independent mechanisms of death.

DNA damage is also a key upstream regulator of different forms of programmed cell death, including apoptosis and necroptosis [47]. Both NaCl and Stx2 are well-known to induce DNA damage while inhibiting the DNA repair processes [48-50]. Throughout our investigation, we observed that co-treatment with NaCl and Stx2 markedly decreased the expression of DNA repair-related genes (*e.g.*, RHNO1, MRE11, HPF1), resulting in augmented DNA damage and augmented G1/S-phase arrest (Figs. 5–7). Interestingly, while Stx2 alone induced caspase-dependent apoptosis, NaCl co-treatment altered the mode of cell death towards a caspase-independent mechanism (Fig. 3E and 3F), potentially encompassing necroptosis, ferroptosis, mitotic catastrophe, or autophagic cell death [47, 51-53]. Pathologically, our data imply that the kidney's distinctive hyperosmolar milieu can increase vulnerability to Stx2-induced injury by facilitating cell cycle arrest and inhibiting DNA repair. Our experiments used an acute exposure model, they do not fully represent the chronic hyperosmotic conditions [54]. However, because DNA repair inhibition continues until cells return to isotonic state [55, 56], chronic hyperosmolarity of the renal medulla can lead to long-term or irreversible damage. Interestingly, clinical experience implies that early hyperhydration therapy, through large quantities of isotonic saline, may alleviate the severity of kidney damage in HUS patients [57]. This implies that osmotic modulation may also be an adjunctive therapeutic tool to prevent toxin-induced renal injury.

In conclusion, this study provides critical insight into how hyperosmotic stress reshapes the cellular response to Stx2 from classical ER stress-induced apoptosis to non-classical, caspase-independent death mechanisms. These findings highlight the paramount importance of considering physiological microenvironments in modeling disease pathogenesis and identifying the unique susceptibility of the kidney to Stx2 due to its native osmotic environment. This work also highlights the importance of adjunctive treatments, such as saline infusion and peritoneal dialysis, not only to preserve fluid balance but also to modulate toxin activity and tissue susceptibility. These results enhance our understanding of Stx2 pathophysiology and provide a foundation for more physiologically appropriate therapeutic intervention in HUS.

## Figures and Tables

**Table 1 T1:** RT-qPCR primers sequence.